# Optical Fiber Sensors by Direct Laser Processing: A Review

**DOI:** 10.3390/s20236971

**Published:** 2020-12-06

**Authors:** David Pallarés-Aldeiturriaga, Pablo Roldán-Varona, Luis Rodríguez-Cobo, José Miguel López-Higuera

**Affiliations:** 1 Photonics Engineering Group, University of Cantabria, 39005 Santander, Spain; pablo.roldan@unican.es (P.R.-V.); miguel.lopezhiguera@unican.es (J.M.L.-H.); 2 Hubert Curien Laboratory, University of Lyon, Jean Monnet University, UMR 5516 CNRS, F-42000 Saint-Etienne, France; 3 Instituto de Investigación Sanitaria Valdecilla (IDIVAL), 39011 Santander, Spain; 4 CIBER-bbn, Instituto de Salud Carlos III, 28029 Madrid, Spain; luis.rodriguez@unican.es

**Keywords:** optical fiber sensor, laser processing, fiber Bragg grating, waveguide, fiber interferometer, cavity, microchannel, reflector, Lab-in-Fiber

## Abstract

The consolidation of laser micro/nano processing technologies has led to a continuous increase in the complexity of optical fiber sensors. This new avenue offers novel possibilities for advanced sensing in a wide set of application sectors and, especially in the industrial and medical fields. In this review, the most important transducing structures carried out by laser processing in optical fiber are shown. The work covers different types of fiber Bragg gratings with an emphasis in the direct-write technique and their most interesting inscription configurations. Along with gratings, cladding waveguide structures in optical fibers have reached notable importance in the development of new optical fiber transducers. That is why a detailed study is made of the different laser inscription configurations that can be adopted, as well as their current applications. Microcavities manufactured in optical fibers can be used as both optical transducer and hybrid structure to reach advanced soft-matter optical sensing approaches based on optofluidic concepts. These in-fiber cavities manufactured by femtosecond laser irradiation followed by chemical etching are promising tools for biophotonic devices. Finally, the enhanced Rayleigh backscattering fibers by femtosecond laser dots inscription are also discussed, as a consequence of the new sensing possibilities they enable.

## 1. Introduction

Since Snitzer proved how optical fibers can behave as a resonant cavity in 1961 [1], a lot of attention and research effort has been drawn to the transducing properties of optical fibers [2,3,4,5,6,7,8]. This led to the beginning of the development of optical fiber sensors (OFS) in the mid-1970s. These new types of sensors exhibited several advantages compared to other sensing schemes, such as electrical immunity, small transmission loss, remote sensing, and high sensitivity, which can outperform other sensors by 10–100 dB in some cases [9]. The initial configurations were mainly interferometric schemes (Fabry–Perot interferometer (FPI), Michelson interferometer (MI), Mach–Zehnder interferometer (MZI), Sagnac interferometer) retrieving a single measurement per sensor (point sensing) [6]. Then, the development of fiber Bragg grating (FBG) technology arisen from photosensitivity studies conducted by Hill et al. in 1978 [10] enabled more compact structures with a higher signal-to-noise ratio. They were marketed in the 1990s and are one of the most widespread OFS schemes thanks to its durability, wavelength multiplexing potential, linear behavior, very small size (few millimeters), and mass production [11].

In addition to point sensing, the demonstration of optical time domain reflectometry (OTDR) in the late 1980s enabled a new branch of OFS known as distributed sensors [12]. Employing Raman, Brillouin, or Rayleigh fiber backscattering, these configurations initially were able to detect a change in the entire fiber with 1-m resolution [13]. Continuous advances on these technologies achieved resolutions as low as centimeters [14]. They are good for long structures such as oil or gas wells and pipelines: this sector accounts for 46% of the total distributed market according to a 2015 market report [15]. Despite the fact that the point-sensing market was larger at the beginning of the century, distributed sensing gained relevance in the next years, matching point-sensors in 2008 and doubling them by 2014 [9].

This market supremacy continues to the present. However, the advancement and application of mature laser technologies allow for more compact structures. New detection parameters in addition to typical temperature, strain, and magnetic field [5] can be measured thanks to new complex structures and specialty fibers [9]. Therefore, an expansion of the industrial and medical market is expected [16]. This is mainly due to an increased effort to conceive new tiny transducer structures that can monitor several parameters, as a Lab-in-Fiber (LIF) [17,18]. This is of special interest in biologic and healthcare applications when probe size can be a major limitation.

In addition, point structures such as FBGs and reflectors can be combined with distributed sensing to offer quasi-distributed sensing with enhanced properties that refine sensing and add additional measurement to key positions [19].

Laser processing began to spread commercially in the 1970s. With 50 years of accumulated experience, this branch of technology offers several possibilities to modify the intrinsic properties of optical fiber. In particular, the maturation of ultra-fast laser technologies offers superb micro-scale change control, vital for truly compact, high-transduction structures [20].

In this work, the most common and enabling structures manufactured using laser processing technologies are reviewed. A development of its foundations and applicability is carried out, as well as a state-of-the-art of current techniques. First, a quick explanation of the basics of laser processing will be tackled. Then, a comprehensive review of the following structures will be performed:

⚬ Gratings: reliable simple and versatile OFSs that has been greatly studied over the last 40 years. Particularly, FBGs are a mature technology relatively easy to produce and replicate being capable to work as sensor, filter and mirror.
⚬ Waveguides: key components for several photonic devices, being essential for photonic integrated circuits (PICs). When writing waveguides into a fiber cladding, they not only allow new sensing configurations, but also the ability to place multiple sensing elements in a single region, significantly reducing transducer length and enabling LIF structures.
⚬ Microcavities: the arrangement of microcavities inside the optical fiber, although it has been traditionally carried out to generate interferometric effects or micro-optical resonators, has begun to gain relevant importance in recent years for its use as a microfluidic reservoir, which allows working together optics and fluidics.
⚬ Microchannels: the main advantage that microchannels provide is that they allow handling of small volumes of biological fluids, which can be interrogated using optical structures. The LIF paradigm naturally integrates these types of structures.
⚬ Weak reflectors: this structure, known as scattering dots, can be manufactured using fs lasers, and they notably increase Rayleigh backscattering. This makes it possible to have fiber sensors with a detection range of up to 100 km, or random fiber lasers, with high discrimination in sensing.

## 2. Direct Laser Induced Optical Effects in Fibers

Laser–material interaction can produce a wide amount of structural changes within a material. These changes are likely to produce modifications in some macroscopic properties. The study of laser irradiation effect on refractive index (RI) of optical materials has been of special interest in the last decades. It can be permanently changed within a broad magnitude range depending on the induced structural modifications that strongly depend on the structural modification. These are triggered by different exposition parameters. In this work, two major types of laser induced refractive index changes (LIRICs) will be considered: the ones induced by the intrinsic photosensitivity of the material, employing UV lasers (continuous and pulsed), and the ones caused by femtosecond lasers.

On the other hand, by using appropiated focused laser beams above damage threshold and hybrid laser-chemical techniques, cavities and channels can be both fabricated in optical components.

### 2.1. UV Laser Beams in Photosensitive Fibers

Among the several advantages of vitreous silica-based glasses as optical materials, it is noteworthy its intrinsic photosensitivity depending on both their composition and exposure conditions [21]. Photosensitivity is a non-elastic light–matter interaction discovered by Hill et al. in 1978 [10] where photoinduced phenomena produce a permanent RI change. Photosensitivity can be caused by several mechanisms dependent on composition; thus, each material requires different light exposition parameters [22]. Here, attention will be drawn towards germano-silicate glasses given its broad application into the core of the fibers and also on Hydrogen (H2) loaded standard telecom fibers.

Photosensitivity in germano-silicate glasses was first associated with the UV light inducing the bleaching of bands attributed to the germanium oxygen-vacancy defect centers (GODCs) band absorption near 244 nm. This band allows single photon absorption exploiting the triplet state of the GODC state at 240–260 nm (5 eV), being able to produce permanent RI change at CW operation. The defects turn into color centers changing its absorption spectrum, thus, changing its RI through Krammers–Kronning relations (KKr). However, this model appeared to give a weak contribution to the RI change [21]. A model better matching the experimental change is the UV-induced densification. This change can be induced by structural changes that lead to a change in polarizability [23]. A number of models employing densification (Clausius–Mossoti equation) as key feature, with its corresponding stress associated (elasto-optic effect) suggest this is the dominant process [24,25,26].

The induced change can be as high as $10^{-3}$. However, this value can be increased up to $10^{-2}$ by a process called $H_{2}$ loading [27]. This method consists of placing the glass in a high-pressure hydrogen atmosphere (40–800 bar) at room or high temperature to diffuse the gas into the glass. This is one of the main treatments for increasing the photosensitivity of germanosilicate glasses due to its efficiency [21].

### 2.2. Femtosecond Lasers

Continuous or pulsed UV lasers, commonly at 244 or 248 nm but also at 193 [28] and 157 nm [29], induce significant RI changes in photosensitive germano-silicate glasses. Femtosecond (fs) lasers, in contrast, given the extremely low pulse duration of ≈100 fs, can easily produce high peak-power densities such as ≈10 TW/cm^2^ where nonlinear ionization effects are produced. This huge irradiance allows multiphoton absorption in silicate glasses allowing permanent RI changes regardless of wavelength and composition. Such changes can be performed with a single pulse [30] compared with the higher exposition times required in UV photosensitivity [23].

#### 2.2.1. Type I RIC

**Figure 1:**
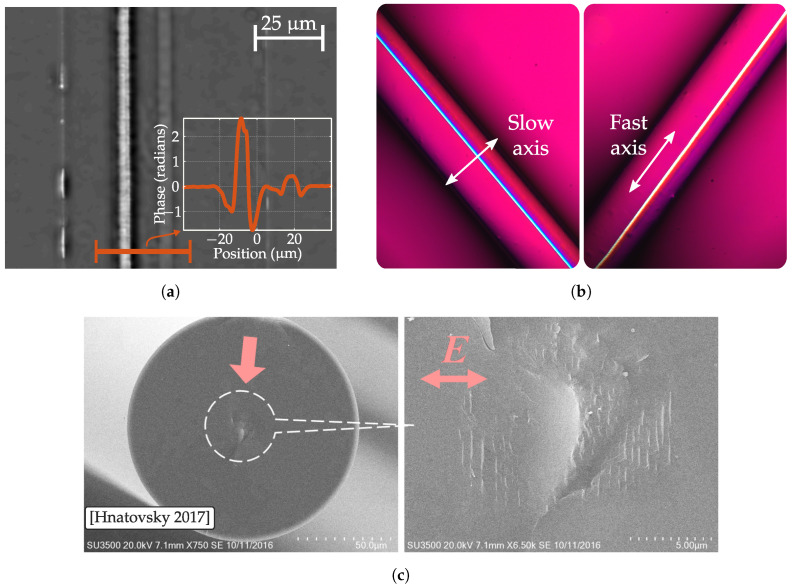
(**a**) Quantitative phase microscopy (QPM) image of an optical fiber inscribed by a femtosecond laser focused 35 μm bellow fiber surface. There is a strong positive Type I stress related modification at core level. Inscription parameters: NA = 0.5, λ=1030 nm, $E_{p}=1.09\;\mu$J, τ = 370 fs, PRR = 120 kHz, and v=10μm/s. (**b**) Transmission optical microscope images of an inscribed optical fiber in an arrangement with a fullwave retarder. The color change reveals the existence of slow and fast axis in the inscribed region; thus, implying birefringence. (**c**) SEM images of a SMF-28 fiber with Type II modifications at core, left figure shows the fiber cross-section and right figure magnifies the core region (adapted with permission from [40], ©The Optical Society).

The first macroscopic effect that fs lasers can produce at relatively low pulse energies ($E_{p}$) (ranging from 0.1 to 0.5 μJ at NA=0.65) is a smooth isotropic refractive index change (RIC) [31]. Its threshold states the first effects associated to optical breaking but below catastrophic breakdown. When this change is manifested alone, it usually exhibits changes ranging from $\Delta n\approx 10^{-4}$ to $\Delta n\approx 10^{-3}$ and when is accompanied by higher types (usually by filamentation process caused by self-focusing) it can reach $\Delta n\approx 10^{-2}$ being this is later often associated to stress deposition. This is the case depicted in Figure 1a where a phase image retrieved by quantitative phase microscopy (QPM) shows an inscription parallel to the fiber core whose phase change (proportional to RIC) is higher than the core.

There is no exact consensus about the mechanism that causes this index change [32]. There are several proposed mechanisms as a function of the material. In the particular case of fused silica, one proposed mechanism is defect formation. Particularly, non-bridging oxygen hole centers (NBOHCs) and E’ centers are though to contribute trough KKr and defect-induced densification to the RIC. However, this cannot be the only mechanism as these defects that would be commonly erased at 200 °C annealing while Type I RIC can resist up to 1000 °C. In fact, these defects increase with the annealing when they are produced by Type I, implying the presence of more underlying rearrangements of the silica matrix [33]. Another alternative explanation is that the electron plasma, when transfers heat to the lattice it quickly heats and cools the affected zone, changing its fictive temperature ($T_{f}$) and hence producing permanent densification/expansion.

#### 2.2.2. Type II RIC

When pulse energy ($E_{p}$) increases above a higher threshold of 0.31μJ (for λ=800 nm, $\tau_{p}=160$ fs, 200 kHz, and NA=0.5 when the irradiated material is fused silica) [34], and several pulses are delivered to the same volume with enough pulse repetition rate (PRR) to interact with each other (When the time between pulses is less than the thermal diffusion time of the material), a large birefringent and negative RIC emerges. It can be as large as ∼$2\times 10^{-2}$ [35] with remarkable thermal stability [36].

An example is depicted in Figure 1b, where an optical fiber inscribed by a fs laser is illuminated by white light in a two cross polarizer and a full wave retarder configuration. Here, the color of the inscription changes with sample rotation. This is caused by an extra retardance whose sign depends on the slow/fast axis orientation with respect to fullwave axes. In this way, the left (right) picture shows the laser inscription when its slow axis is parallel (perpendicular) to the slow axis of fullwave retarder. Thus, manifesting its birefringent nature.

The induced birrefringence is not related to stress as in Type I but to the formation of sub-wavelength features of the so-called nanogratings or nanoplanes. These structures are composed of several nano porous layers perpendicular to polarization direction where ${\mathrm{SiO}}_{2}$ releases oxygen atoms through bond breaking [37]. The grating arrangement is thought to be caused by an interference mechanism between pulse and some kind of seed element. These seeds have to be generated by multiple pulse depositions as nanograting formation requires several pulses per micrometer (from 10 to 100 pulses) [38]. Beresna et al. propose that polaritons are created from multiphoton absorption interfere with self trapped excitons creating the nanograting pattern [39]. These excitons relax into defect formations that eventually leads to oxygen emigration [38]. Figure 1c depicts the work of Hnatovsky et al. [40], two SEM images of a standard single mode fiber (SMF-28) fiber with Type II modifications at core. Left figure shows the fiber cross-section and right figure magnifies the modified portion where nanogratings perpendicular to polarization axis are clearly seen.

### 2.3. Femtosecond Laser Irradiation Followed by Chemical Etching

In recent times, there has been a notable increase in the development of lab-on-chip (LOC) platforms; that is, hybrid microsystems that combine functionalities that would involve a complete biological laboratory [41,42]. Typically, they consist of networks of microfluidic channels that are located on a substrate and optical waveguide structures [43,44,45,46]. In this way, it is possible to integrate numerous sensor elements in a small space and thus have great flexibility in handling small volumes (μL, nL) of biological fluids [20,47]. That is why this type of platform has great applicability in fields such as biology (e.g., genomics), chemistry [48], or even clinical procedures.

A variant of LOC platforms are LIF devices. They allow combining the advantages of LOC sensors in optical fibers [8,49], knowing that fused silica is highly favorable for biocompatibility. The combination of optical fiber and microchannels allows working together with optics and fluidics (*optofluidics*) [41,50]. Thus, the presence of the fiber core as a waveguide allows the different optofluidic sensor components to be interconnected optimally, being able to optically interrogate the microfluidic elements.

A widely used approach to the creation of three-dimensional structures in optical fibers is the fs laser. It offers great simplicity and flexibility when inscribing patterns directly inside the bulk volume of transparent materials [51,52]. Through the refractive index changes induced in the material, it is possible to create optical waveguides, lenses, or more complex structures such as splitters or interferometers, among others [20]. Additionally, fs lasers also offer the possibility of directly manufacturing microfluidic channels, using the femtosecond laser irradiation followed by chemical etching (FLICE) technique [41], which is detailed below.

**Figure 2:**
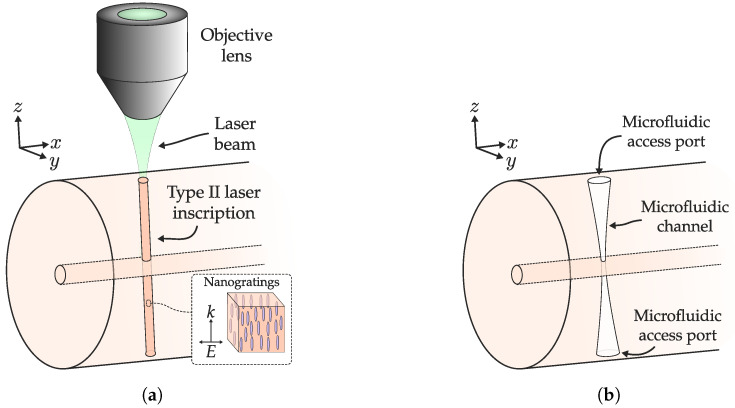
Illustrative diagram of the formation of microchannels in fused silica (optical fiber) using the femtosecond laser irradiation followed by chemical etching (FLICE) technique. (**a**) Step 1: Permanent Type II modification in the sample by non-linear absorption of focused femtosecond laser pulses. (**b**) Step 2: Chemical etching of the laser-modified region generates a microfluidic channel with a given aspect ratio.

As shown in Figure 2, the manufacture of microchannels in fused silica, as well as in general substrates, is based on two clearly differentiated stages: (1) processing of the material with the femtosecond laser, (2) and chemical etching of the processed area for the formation of the microchannel. Sometimes an additional step is performed between both processes, which corresponds to a thermal annealing of the structure [50]. This allows smoothing laser-induced modifications (less roughness), which favors acid diffusion throughout the processed area.

Regarding fs laser processing, it is important to highlight that, unlike the manufacture of waveguides or other optical structures, where smooth, uniform, and positive refractive index changes are intended (Type I); in the first step of the FLICE technique, Type II modifications are preferable. This does not imply that Type I changes cannot be used, since with both types of RIC the etching selectivity is increased after femtosecond laser irradiation. However, although with Type I modifications the increase is very weak, with Type II RIC the etching rate can improve up to two orders of magnitude compared to pristine fused silica. In the latter case, the improvement in etching rate is fundamentally due to the formation of self-ordered sub-wavelength nanogratings. These nanoplanes, with a ∼λ/2n period (λ is the fs laser wavelength, *n* is the RI of medium), tend to be coherently aligned (with adequate fluence in the focal volume), and are oriented perpendicular to the laser polarization direction Figure 2a) [38,53].

From the above, two characteristics of the FLICE technique stand out. First of all, it is a multi-pulse effect. According to multiple proven works, to achieve a Type II RIC, the interaction of two or more pulses is required (typically they are usually more than 10) [38]. Hence, parameters such as inscription speed, laser PRR, or pulse energy have to be chosen appropriately. The second important point is the relationship between the laser inscription and polarization directions [54]. Considering that the nanogratings are perpendicular to the polarization direction, if polarization and inscription directions are perpendicular, the nanogratings will be oriented parallel to the microchannel axis. In this way, the acid diffusion along the channel is favored. Otherwise, the nanoplanes block diffusion, preventing the formation of the microchannel.

Therefore, step 2 of the FLICE technique can be understood as the fast diffusion of the acid favored by the nanogratings of the irradiated area that causes etching of the fused silica along the diffusion path. Using HF as an acid, the chemical reaction that causes etching of fused silica is ${\mathrm{SiO}}_{2}+4\mathrm{HF}\to {\mathrm{SiF}}_{4}+2H_{2}O$ [41] in which the etching rate, is temperature dependent. In the process, it is necessary to remove the reaction products and refresh the acid so that it diffuses in the depth of the etched microchannel. However, if the channel is long, it is difficult to refresh the acid at the end, and therefore the etching rate decreases. That is why microchannels with conical shapes appear, wider in the microfluidic access port, and narrower away from it (Figure 2b). Ultrasonic baths are usually used in chemical etching, in order to promote acid diffusion and achieve microchannels of greater length and aspect ratio (length to diameter ratio) [55].

Typically, there is a trade-off between microchannel aspect ratio and length. High HF concentrations produce channels of greater length, but with a smaller aspect ratio (faster lateral etching in access ports) [55]. On the other hand, low concentrations lead to shorter channels, but with high aspect ratios [56]. As an alternative to using HF as a chemical agent for selective etching of fs laser irradiated microchannels, KOH solutions have recently been employed [57,58]. Despite their long etching time, they produce markedly longer microchannels than with HF solutions. Later, in the development of microchannels as a sensor structure, the characteristics of the microfluidic channels achieved in the literature will be studied.

## 3. Optical Structures and Applications

### 3.1. Gratings

Gratings are small perturbations induced in the fiber with a given period Λ that decouple or reflect the light of fundamental mode with a spectral dependence on Λ. This means, the reflected and/or transmitted grating spectrum will exhibit resonance peaks sensible to the grating period. Any grating deformation as a consequence of a perturbation, e.g., temperature, strain or pressure, will produce a peak shift that can be employed for sensing. Gratings are reliable, simple, and versatile OFSs that have been greatly studied over the last 40 years.

**Figure 3:**
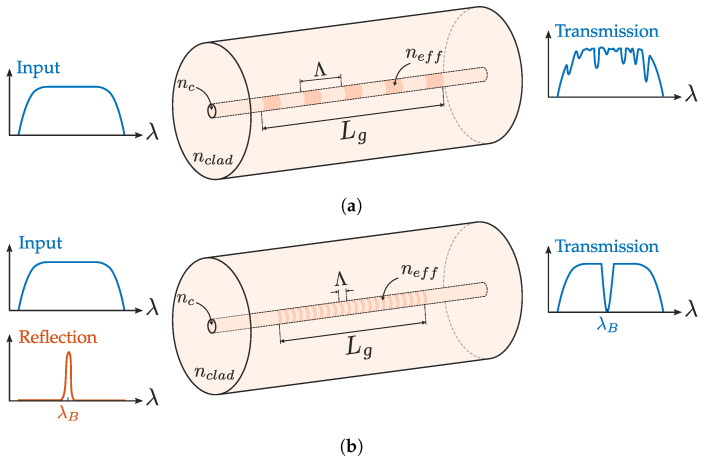
(**a**) Gratings with a period Λ≫λ are known as Long Period Gratings (LPG) and couple the light of fundamental mode to the cladding modes. (**b**) When the grating exhibits lower periods Λ≤λ, it reflects light at Bragg resonance and is known as fiber Bragg grating (FBG).

Gratings can be divided into two types depending on Λ. For periods higher than wavelength Λ≫λ, the grating is known as Long Period Grating (LPG) and decouples light from fundamental mode to cladding modes following the well known coupled-mode condition [59] (depicted in Figure 3a). Given their long period, there are several ways to inscribe LPGs [60]. Laser processing techniques usually involve CO${}_{2}$ lasers, UV lasers with photosensitive fibers and ultrafast lasers. These structures can be employed in several sensing applications as the main transducer. In addition, they can be employed in more complex devices, such as fiber interferometers [7] or can enhance the response of another transducing structures, such as the SPR of a coating [61].

This work will focus on the second type of gratings depicted in Figure 3b, i.e., FBGs. These gratings exhibit periods in the same order as wavelength (Λ∼λ), and they operate in Bragg regime. Thus, they reflect the fundamental mode at a given wavelength following the Bragg condition

(1) $$\lambda_{m}=\frac{2}{m}n_{eff}\Lambda$$

where $n_{eff}$ is the effective index of fundamental mode and m an integer representing the harmonic order of resonance wavelength, which is related to the harmonic decomposition of the periodic pattern.

FBGs are a mature technology relatively simple to produce and replicate being capable to work as sensors, filters, and mirrors. As sensors, they are one of the most commercially spread point OFS (only surpassed by fiber gyroscopes [16]) and there are several types and methods to manufacture them.

#### 3.1.1. Dependence on Inscription Source

First, FBGs were inscribed in photosensitive germanium or boron-doped fibers by UV laser sources and several techniques that generate an interference pattern in the fiber core. This pattern can be achieved by several interferometric methods using two coherent UV beams (including the phase mask technique). In this way, the result is a highly sinusoidal pattern inscribed in the fiber core with an index change ≈10^−4^ that can be increased up to ≈10^−3^ with hydrogen load techniques [62]. These FBGs can typically operate up to 80 °C for 25 years [63]. Reflection and FWHM of the reflected peak exhibit remarkable behavior. Unfortunately, as mentioned in Section 2.2, they can only be inscribed in the core of photosensitive fibers. These laser sources are unable to write in the cladding. For this reason, fs lasers are also employed in FBG inscription. To understand the main differences between both writing sources, Table 1 depicts a grating classification in function of UV or fs-IR inscription.

There are three main types of FBGs: Type I, whose inscription is below damage threshold; Type II, which are written above damage threshold ( do not mistake with aforementioned types in Section 2.2, theses are general categories to also cover UV laser modifications); and Type R or regenerated gratings.

Among Type I, there are several subcategories such as hydrogen loaded gratings (Type IH), negative index gratings (Type In), or densification gratings (Type Id). In this work, the attention will be focused on standard Type I UV and Type I fs-IR gratings, which are the UV photosensitive and femtosecond written, respectively; both with smooth RICs. For an extended list of Type I gratings, the reader is referred to [66]. Type I UV gratings are completely erased at 600–800 °C, while Type I fs-IR can work up to 1000 °C [64]. The latter is produced by Type I modifications below the damage threshold. Thus, it is a smooth and homogeneous refractive index change that exhibits good spectral quality similar to its UV counterpart. However, from a thermal point of view, these gratings exhibit thermal stability more similar to Type II gratings due to the increased resistance of Type I modifications. This also leads to some ambiguity when classifying an fs FBG into Type I or Type II.

Type II UV FBG is written by a single pulse of an excimer laser during the fiber drawing process. The high exposure dose surpasses damage threshold of the photosensitive fiber. As a result, the fiber exhibits permanent photoinduced damage [68,69], that is much more resistant to ultra-high temperatures, but with higher scattering losses and lower repeatability. With femtosecond lasers, FBGs can be achieved by direct-write technique when a strong Type I modification and/or microvoids are formed. The phase mask is also employed, producing Type II modifications. These structures exhibit an inhomogeneous refractive index change and also scattering losses. Moreover, self-focusing often takes place. Given the permanent behavior of these structures, they can withstand 10 h at 1200 °C [67], exhibiting remarkable stability. However, due to the heterogeneity of these modifications, the regions with soft RIC below damage threshold might be erased. This may cause some fluctuation in the grating transmission/reflection with temperature if not conveniently annealed. As Type I modification can produce both Type I and Type II fs-IR gratings (depending on the modification strength, the thermal behavior and shape of the structure), specification of modification type in addition to grating type is strongly suggested.

The peculiar third category contains the FBGs achieved by the regeneration process. Regeneration involves the complete erasure and resurrection of a seed FBG (both Type I UV or Type I fs-IR) through thermal annealing. The seed structure changes the thermal history of glass while it is erased, which modifies the local response to homogeneous thermal processes. This response consequently produces different parameters in processed and unprocessed regions. Thus, the fiber achieves a fictive temperature modification of a similar period that acts as a new FBG [70]. The regenerated FBG can work up to 1295 °C with comparable losses [63,66].

#### 3.1.2. Direct-Write Inscription

**Figure 4:**
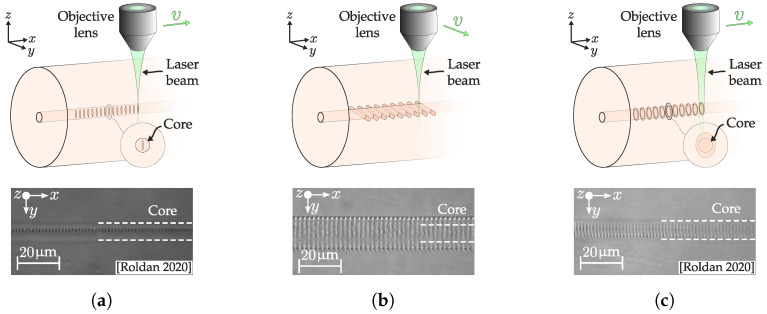
(**a**) Point-by-point inscription scheme and microscope image of the resulting FBG extracted from [83]©IEEE. (**b**) Line-by-line inscription scheme andmicroscope capture of a typical FBGmanufactured with this technique. (**c**) Plane-by-plane (Pl-b-Pl) inscription scheme and Pl-b-Pl grating inscribed with same writing setup as in (**a**) but with an slit of 1mmwidth abovemicroscope lens (extracted from [83]©IEEE). All the samples weremanufactured at the Photonics Engineering Group, Santander, Spain.

In addition to higher temperature resistance, the writing potential of fs lasers enables different inscription methods apart from phase masks and other interference techniques. Those are collected within direct writing methods which inscribe one modification at a time instead of all the periodic modifications at once as the phase mask does. These methods attract much attention due to their intrinsic versatility [71]. They can be divided into three different types:

- Point-by-point (PbP): Each laser pulse or pulses are delivered in the fiber with spacing Λ. The simplest way to achieve this involves a moving platform that displaces along the fiber axis with a velocity v=Λ·PRR. In this way, each pulse is deposited with a homogeneous spacing of Λ as depicted in Figure 4a. Unlike the phase mask, the resulting index modulation exhibits a sharper pattern than a perfect sinusoidal. The pattern can be assumed as a Fourier series whose 2nd to, at least, 4th harmonics are non-negligible. Thus, the light also interacts with them and is reflected at wavelength proportional to the harmonic period [72]. This greatly simplifies FBG inscription as higher periods can be inscribed, producing good results. In addition, the modification cross-section depends on the focal volume. In transversal writing, given the mismatch between the Gaussian Rayleigh range and the neck waist, the resulting pattern is not perfectly circular and is usually [73] smaller than the core. Thus, the modification cross-section is not uniform, which has two major implications. First, the coupling to cladding modes is increased [74], exhibiting a high number of peak resonances that can even overlap sometimes [75]. Second, the fundamental mode propagates through an asymmetrical structure, raising the grating birefringence and the polarization dependent losses (PDL) [76]. This last issue has been addressed with specific adaptive optics arrangements [77].
- Line-by-line (LbL): Instead of a single point, a transversal sweep to the fiber axis produces a line as depicted in Figure 4b. Each line is inscribed with a spacing Λ that determines the grating period. This method was first reported by Zhou et al. [78], achieving stronger Bragg resonance, lower insertion and polarization dependent losses than PbP. In this way, more core surface interacts with the grating, producing a more homogeneous index modification that reduces the coupling of cladding modes coupled. This larger and also smoother area can allow lower broadband losses, which depend on the length and roughness of the lines. This spectral improvement combined with the grating structure makes them suitable for tilted inscription. Furthermore, the LbL inscription method allows the manufacture of relatively complex structures, by allowing flexible control over each inscribed line (e.g., induced refractive index change [79]). The main drawback of this inscription technique is its slow speed. PbP is remarkably faster and can write a grating in a few seconds while LbL needs some minutes for the same length.
- Plane-by-plane (Pl-b-Pl): The next step involves the inscription of a quasi-homogeneous planar structure that covers the entire core cross-section. This methodology was first achieved by Theodosiou et al. [80] performing single sweeps with careful control of width and depth parameters. The result exhibits greater spectral quality than PbP and LbL, with lower FWHM and broadband losses. This method can be improved by properly shaping the beam into a plane that covers the core region (Figure 4c). This arrangement significantly increases the quality and inscription speed of the grating. Effective beam shaping has been performed by Lu et al. by means of a cylindrical lens that induced that induces astigmatism in the focal volume [81]. Another beam shaping method that can produce similar results is the slit beam technique developed by Ams et al. [82]. Here, a single slit increases the waist of the focal volume in the transverse direction of the fiber (*Y* direction in Figure 4c). This method achieves a spatially wider and more circular plane. This improvement can be clearly seen in Figure 4a,c where transmission microscope images of a FBG written without and with slit are respectively depicted. The reported FBGs written with this technique exhibit lower PDL, FWHM, broadband losses, and higher reflectivity than its PbP counterpart at the same effective pulse energy [83].

#### 3.1.3. Configurations and Applications

Fiber Bragg gratings, as a mature technology, have been extensively studied and a wide set of structures and their applications can be found in the published literature. In this work, attention will be focused on tilted gratings, polarization-dependent gratings, and slow light.

Tilted gratings: When the planes of the gratings are written with certain angle respect to the propagation direction, the energy coupling to the counter-propagating fundamental mode is reduced but also, the counter-propagating cladding mode coupling is enabled. This interchange is produced when the mode wavelength follows the couple mode condition:

(2) $$\lambda_{clad,i}=\left(n_{eff,core}+n_{eff,clad}\right)\frac{\Lambda }{\cos\theta }.$$

**Figure 5:**
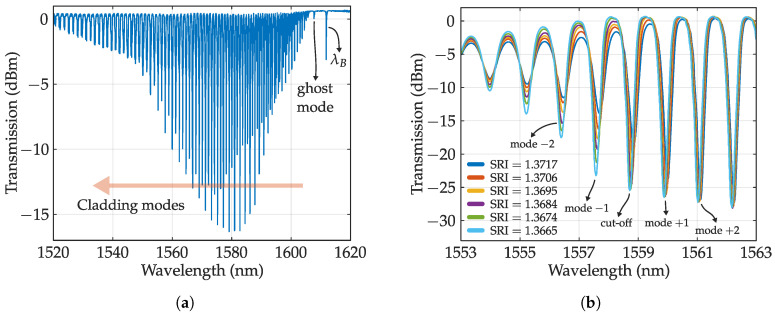
(**a**) Conventional transmission spectra of 6° tilted FBG (TFBG). (**b**) Near “cut-off” cladding mode resonances with different refractive index (RI) immersions. Both are reproduced from [87].

Thus, the cladding modes will be reflected in a spectral comb located at the right of resonance wavelength. Each cladding mode exhibits different propagation constant. It is noteworthy the mode next to $\lambda_{B}$, called ghost mode that shares some properties with fundamental mode. The complete transmission spectra is depicted in Figure 5a. The coupling is produced over a wide spectral range with little tilt angle required, over 100 nm for a tilt angle of 10°. The coupling is also polarization selective, being TM/EH transversal modes coupled by P-polarized light and TE/HE by its counterpart. The tilted pattern can be written with a conventional phase mask and UV laser, however, it requires a strong RI modulation. This issue is usually fixed by increasing the photosensitivity of the optical fiber by methods such as hydrogen load. The strong RIC of fs irradiation can appropriately write tilted FBGs (TFBGs) with phase mask [84] or LbL [85] and Pl-b-Pl [86] methods. The highly resolved cladding modes are sensitive to bending and also refractive index, which are usually insensitive parameters for the Bragg wavelength. Particularly, when the optical fiber is surrounded by a medium whose RI is higher than the effective index of a certain cladding mode, that mode and lower modes will be radiated out from the fiber. Thus, the spectrum consequently suffers an attenuation in the upper envelope of the cladding modes up to the “cut-off” mode. In addition, the cut-off mode suffers a wavelength shift due to the evanescent wave penetrating the outer medium. This shift and attenuation are depicted in Figure 5b. The cut-off mode can exhibit sensitivities of 25 nm/RIU and resolutions of the order of $10^{-4}$ which is not outstanding [87], but with the proper combination with coatings (exploiting plasmon resonance), sensitivity can be increased up to 500 nm/RIU [88,89].

**Figure 6:**
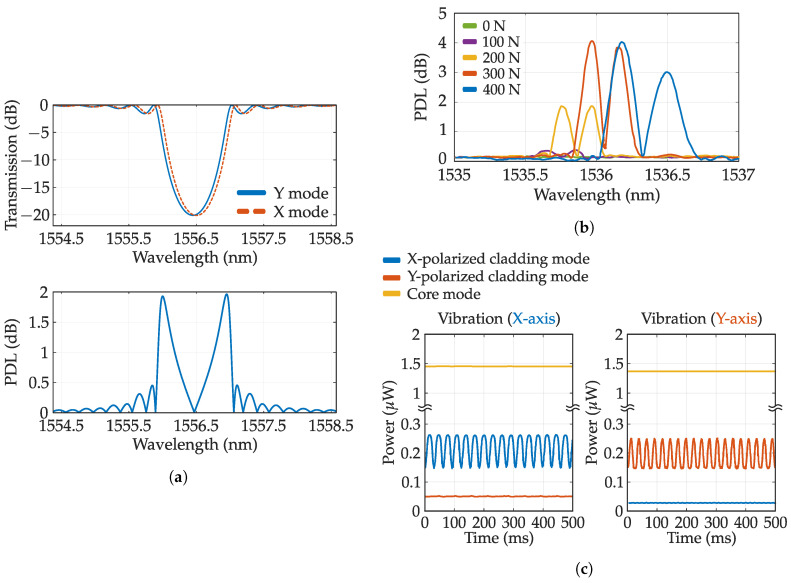
(**a**) Transmission and polarization dependent losses (PDL) spectra for a uniform FBG (parameters used for the simulation: *L* = 2 mm, Λ = 538.1 nm, and $\delta n=10^{-3}$ (**b**) PDL evolution as function of the transverse force (extracted from [76]). (**c**) Cladding mode amplitude of an FBG written in a PM fiber against time. Vibrations parallel to the polarization state affect cladding mode amplitude while perpendicularly polarized light is insensitive (extracted from [92]).

Polarization assisted FBGs: An ideal optical fiber can be regarded as an isotropic medium. However, any bending or asymmetry in the structure may produce a small birefringence. This anisotropy can also be produced when an FBG that breaks the circular symmetry of the fiber is written. Birefringence produces different transmission values depending on polarization. PDL are the maximum change between all these states and are depicted in Figure 6a with a simulation performed by the module GratingMOD of commercial software RSoft. The top figure depicts the shift between TE and TM polarization and its resulting PDL. These losses can be used for sensing parameters that are spatially oriented, such as lateral force, bending, or twisting, and also for measurements of the properties of anisotropic media. In addition, this value can be used in addition to the regular grating spectra to perform multi-parameter sensing.

Lateral force, for example, can be measured in regular FBGS by measuring the spacing between orthogonally polarized resonances, which can be clearly seen in a PM fiber [90]. However, PDL, as is proportional to birefringence, can also be employed. Figure 6b depicts the PDL evolution as function of the transverse force where peak amplitude increases with applied force (extracted from [76]). The amplitude behaves linearly with transversal force up to a critical point that depends on the grating length among other parameters. PDL exhibits more sensitivity without requiring PM fibers which are also dependent on loading angles [76]. Twist can also be measured with the PDL spectrum of a homogeneous FBG by measuring the ratio between the two main lobes [91]. The sensitivity can be further increased with a PM fiber. This is shown in Figure 6c where cladding mode amplitude of an FBG written in a PM fiber is depicted against time. Vibrations parallel to the polarization state affect cladding mode amplitude while perpendicularly polarized light is insensitive [92]. In this way, 2D sensing is achieved.

Bending compresses the fiber region close to the inside bending radius while stretches its outside counterpart. Therefore, any symmetric region compensates the stretched region with the compressed region resulting in a neutral average modification. This compensation makes $\lambda_{B}$ of FBGs inscribed at core insensitive of bending (unless the core is already birefringent as in PM fibers [93]). Some configurations translate fiber bending into measurable strain, but they require additional elements attached to the optical fiber [94]. However, because bends introduce significant RIC of opposite sign due to compression and tension of the fiber, the shape and effective index of cladding modes are perturbed. Thus, their resonance wavelength and coupling strength will change with bending. Cladding modes can be produced with an asymmetric FBG, also known as Eccentric FBGs and aforementioned TFBGS. Both grating types can measure bending without PM fibers [95,96,97,98].

**Figure 7:**
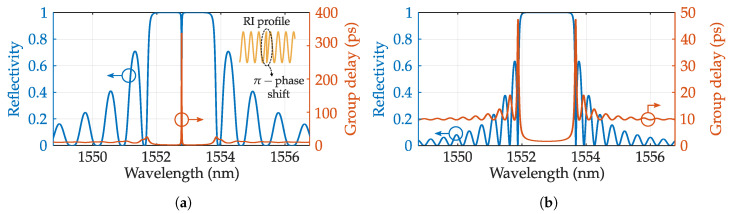
(**a**) Reflectivity and group delay of a 2 mm length p-phase grating. (**b**) Reflectivity and group delay of a 2 mm length slow-light grating.

Slow light: In a conventional grating, the group delay increases for wavelengths in the vicinity of the resonance bandwidth. This can be understood as the light being reflected within the FBG, enabling structural slow light at certain resonances. These resonances exhibit higher sensing potential than Bragg resonance due to the extremely narrow bandwidth. An ideal FBG exhibits a narrow reflection peak at expenses of low reflectivity unless it has an unreasonable length. Slow light resonances can exhibit 15 pm bandwidth with only 2 mm length [99]. One way to achieve such resonances is the π-phase gratings. These structures exhibit a π phase shift in the middle. In other words, the grating is composed of two consecutive gratings of the same length and a phase difference of half λ. The resulting RI modulation is shown in Figure 7a with a typical reflection and group delay pattern simulated with the module GratingMOD. Here, each grating act as a reflector forming a single mode Fabry–Perot. The resonance is located at the middle reflection peak and significantly narrow. The phase shifted gratings are commonly writing with a phase mask [100], but there are also works where LbL inscription has been employed, featuring twist sensors with sensitivity up to −1032.71 dB/(rad/mm) [101]. These gratings can perform multi-parameter sensing when inscribed in PM fibers, allowing high resolution in two parameters simultaneously [102].

Another way to achieve slow light resonances is through a strong uniform FBG. These gratings produce sharp lobes in the vicinity of the resonance peak that corresponds with light partially reflected and transmitted, acting again as a high finesse FP cavity. A typical reflection pattern is depicted in Figure 7b where group delay shows the slow light resonances. This method can be improved by properly apodizing the grating [103]. In the same way as π-shifted FBGS, these slow light gratings can act as extremely sensitive sensors when properly combined with advanced interrogation techniques [104,105].

### 3.2. Cladding Waveguides

Waveguides are key elements for several photonic components, being essential for photonic integrated circuits (PICs) which are usually manufactured by means of UV lithography [106,107].

Optical fibers are usually manufactured by drawing an existing preform [108]. These fibers commonly exhibit a single core. However, there are several scenarios where multiple cores are desired, such as Space-division multiplexing (SDM) [109] and sensing. Multicore fibers can be manufactured by similar technologies than SMF such as the stack and drawn process [110]. Alternatively, the microstructured hole arrangement of Photonic Crystal Fibers (PCFs) can also be applied [109]. These fibers, when spliced and fused with common optical fibers can behave as sensors [111]. However, multicore fibers are expensive, especially when a specific configuration is desired. In addition, they have coupling issues and are not adequate for very short lengths (<5 mm). Here, cladding waveguides (CWG) inscribed by fs lasers [112] offer a versatile alternative that can be manufacture on-demand regarding the specific needs. These waveguides are of special interest as they can be employed for decoupling light from the fiber core and couple it to other cladding transducer structures such as FBGs [113,114]. This waveguide not only allows new sensing configurations but also the possibility to place several sensors in a single region, significantly decreasing sensor length and enabling LIF structures [18].

#### 3.2.1. Waveguide Types

**Figure 8:**
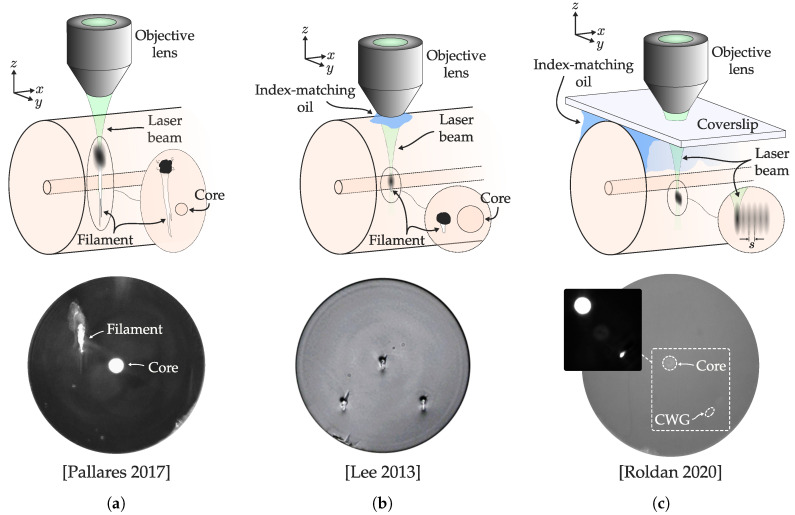
(**a**) Schematic of filamented waveguide. High negative refractive index change (RIC) at focal volume with guiding filament below (extracted from [118], ©IEEE). (**b**) Immersion oil microscope inscription. More controlled filament (adapted with permission from [121], ©The Optical Society). (**c**) Adaptive optics with multi scan technique. Smooth RIC, controlling width which each scan (extracted from [122], ©Elsevier).

There are several works reporting waveguides inscribed by femtosecond lasers in bulk glasses [20,33,82,115,116,117]. However, the number of studies reporting waveguides inscribed in optical fibers is significantly lower. The coupling difficulties, the inscription size, and of course the cylindrical geometry of the fiber greatly hinders the inscription compared to bulk glass. Table 2 depicts a list of inscription parameters of reported cladding waveguides which can be categorized depending on the method of addressing the wavefront distortion caused by the cylindrical geometry of optical fibers. First, there are filamented waveguides that do not correct the aforementioned geometry. These waveguides employ high pulse energies to produce Type II modifications and filamentation effect. Figure 8a depicts a schematic and a microscope image of a typical cross-section with the negative part and the filament below. This later region exhibits a high positive index change (∼10${}^{-2}$) [118] that can be easily employed as a waveguide. This is a simple and straightforward method to achieve waveguides; however, the resulting guiding structure is highly asymmetric, exhibiting high PDL and coupling losses to the fiber core. Among the total modified region, only a small section guides the light while the remaining regions are unemployed, limiting the number of waveguides inscribed in the same region. In addition, the inscription of Type II reduces the mechanical resistance of the fiber and can produce high transmission losses. Another writing option involves an oil immersion microscope to address the cylindrical lens effect [119,120]. In this method, a more controlled cross-section is achieved as depicted in Figure 8b. The shorter guiding filament improves the mode symmetry. This method produces robust results with a wide range of PRRs. Usually, an increase in PRR involves an increased amount of pulses per length unit. When this happens, pulse energy must be reduced to avoid optical damage in the waveguide.

The waveguide cross-section can be further controlled by employing the multiscan technique. This method involves the cross-section control through several scans performed with a small displacement each as depicted in Figure 8c. This technique employs a simple adaptive optics arrangement employed by Zhou et al. [123] consisting in the optical fiber surrounded in index matching liquid, sandwiched between a microscope slide and a coverslip. This arrangement compensates astigmatism caused by the cylindrical geometry, producing significant changes in not only the shape but also the inscription properties [73]. The modified region is lower than in previous methods but with a homogeneous and smoother RIC. In this way, the cross-section can be controlled by the number of scans performed. In addition, this method modifies the refractive index change with each scan up to a limit, which allows increased control over waveguide properties [122,124]. This technique has been employed to manufacture remarkably complex sensors [112].

There is plenty of room for improvement in these reported waveguides and several inscription techniques demonstrated in bulk should be employed. Especially, those techniques allowing symmetrical waveguides are of special interest to improve the coupling/decoupling to the fiber core and reduce waveguide polarization losses. For example the astigmatic beam shape [116] and slit width technique [82] could be employed to improve the waveguide response.

#### 3.2.2. Some Applications

**Figure 9:**
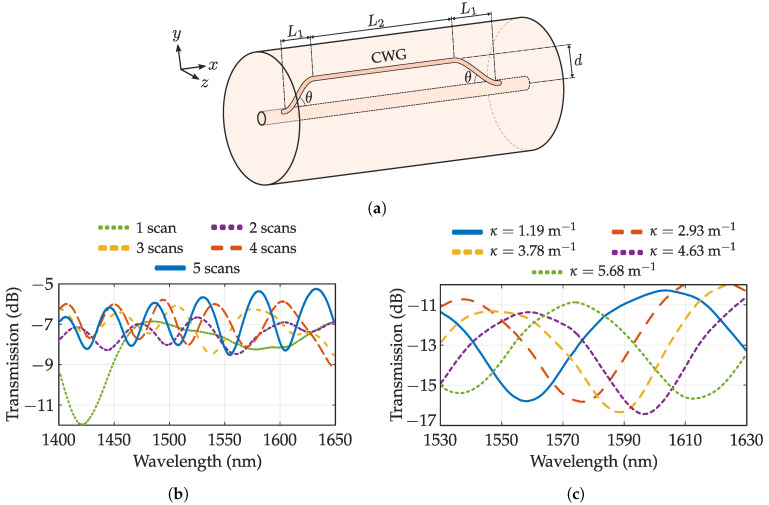
(**a**) Schematic of cladding waveguide Mach–Zehnder interferometer (CWGMZI). (**b**) Different MZI spectra inscribed with the multi-scan technique with different number of scans [122]. (**c**) Spectra of an MZI bent with different curvatures. The spectra exhibit a red shift [118].

As already mentioned, cladding waveguides exhibit a tremendous interest for several sensing schemes. Here, some direct applications will be highlighted. The simplest transducing structure achievable with cladding waveguides is a Mach–Zehnder interferometer (CWGMZI). This device consists of a CWG decoupling light from the core, serving as reference arm to couple it back to the core with an optical path difference proportional to the interferometer length and effective index difference between core and waveguide. This arrangement is depicted in Figure 9a where the aforementioned path is highlighted. The operating principle is based on the well-known interference equation:

(3) $$I=I_{1}+I_{2}+2\sqrt{I_{1}I_{2}}\cos\left(\delta_{MZI}\right),\;\;\;\delta_{MZI}=\frac{2\pi (\Delta n)L}{\lambda }+\phi_{0},\;\;\;FSR\approx \frac{\lambda_{0}^{2}}{\Delta n\cdot L}.$$

where $I_{1}$ and $I_{2}$ are the core and CWG irradiances, respectively. $\delta_{MZI}$ is the optical path difference (OPD) of the MZI when the core and CWG length difference is neglected. Some typical CWGMZI spectra are depicted in Figure 9b where the multi-scan technique has been employed. Here, spectra exhibit a sinusoidal behavior as expected from Equation (3) where dip distance, also known as Free Spectral Range (FSR), decreases with the number of scans (which increases Δn). The amplitude of interference dips strongly depends on the amount of light coupled to the CWG being 50:50 coupling ratio the maximum interference achieved. For this reason, the CWG geometry at both the beginning and end of the interferometer are carefully selected to best balance the coupling ratio. In his regard, Zhang et al. employed a coreless fiber to achieve symmetric coupling between laser-written core and cladding waveguides [119]. The interference dips shift when a perturbation on the sensor length or an asymmetric index change in the fiber is produced. In this way, CWG MZIs have been employed as temperature, strain RI, and curvature sensors as depicted in Table 3. The temperature has been extensively studied, reporting sensitivities ranging from 23–490 pm/°C, which is higher than an FBG but with lower dynamic range. The huge sensitivity range is attributed to the different writing parameters as the dip shift is caused by an RIC in the CWG. From a previous study where the CWG MZI was exposed to high-temperature [125], a non-linear behavior for temperatures above 180 °C is observed with a dip amplitude decrease (suggesting he erasure of the CWG at those temperatures).

Axial strain and bending are the other two parameters that can be measured with superior sensitivity than an FBG. Particularly, the bending exploits the induced birefringence of the fiber to change the effective index of the CWG arm while the core index remains constant [126]. This behavior is depicted in Figure 9c where interference dips are shifted with a curvature increment. The bending sensitivity and dynamic range are remarkable for its short length, however, bending sensitivity is strongly dependent on bending direction and the spacing of CWG from the core. In addition, it can interfere with strain measurement. This cross-sensitivity can be easily fixed by another sensing element inscribed in the interferometer. There are works reporting an FBG inscribed in both CWG [112] and core [122] of the MZI, increasing its sensing potential.

Surface refractive index change, like bending, are sensing parameters that highly depend on the CWG position. High proximity to the cladding/surrounding allows high sensitivity to index changes while proximity to the core makes it insensitive. The evanescent wave of the CWG is enhanced as the RI of the surface approaches that of the fiber cladding. In this way, the dip shifts exponentially with the index change achieving high sensitivity ∼300 nm/RIU when RI is ∼1.432 in the work reported by Zhang et al. [119].

**Figure 10:**
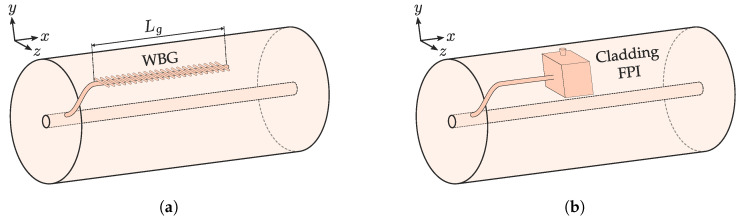
(**a**) Schematic of a BWG. (**b**) Schematic of a Cladding Fabry–Perot Cavity connected through a waveguide.

Other reported applications of CWG are the WBG, inscribed waveguides with an FBG. Light from the core is decoupled in a similar way than the CWGMZI but with an FBG inscribed in the waveguide and no return arm (Figure 10a). In this way, the FBG becomes sensible to index change [113], and bending. Unlike other curvature sensors, the asymmetric position of the FBG also is sensible to bending direction. Waltermann et al. exploited this property to 3D shape sensing the optical fiber by multiplexing several WBG over a 10 cm region [114].

Cladding waveguides can couple to several other transducing elements besides FBGs. It is noteworthy the contribution of FLICE to generate several structures that can be through CWGs. Haque et al. reported FLICE cavities such as Fabry–Perot interferometers (FPI) connected to the core through a CWG in a similar way than Figure 10b [8].

### 3.3. Microcavities

The microcavities that can be generated in an optical fiber can be used in many applications, although it should be noted that they are typically used as interferometers, in some of their different configurations. However, they can also be used as micro-optical resonators [8,127,128], microfluidic reservoirs [8], or even as LPGs [129].

Next, the in-fiber microcavities interferometric operation principle will be detailed, as well as the different laser manufacturing methods that exist. Finally, some notable sensor structures existing in the literature will be exposed.

#### 3.3.1. Operation Principle

As discussed in the waveguides section (Section 3.2), interference occurs due to the superposition of two or more electromagnetic fields that differ in the phase due to an existing OPD. The resulting intensity can be represented by a more general equation

(4) $$I=I_{core}+\sum_{i}I_{sec}^{i}+\sum_{i}2\sqrt{I_{core}I_{sec}^{i}}\cos\left(\delta_{i}\right),$$

where $I_{core}$ and $I_{sec}^{i}$ are the light intensity of the core mode, and the *i*th secondary mode, respectively; $\delta_{i}$ is the phase delay between the core mode and the *i*th secondary mode. Unlike what happened in CWGMZI, when using in-fiber cavities, the interference pattern is mainly formed by the core and cladding modes, which is why multiple secondary modes have to be considered.

Regarding the phase delay, its value depends on the interferometer configuration used. Using in-fiber cavities it is possible to make MZIs, MIs, and FPI. Although $\delta_{i}$ depends specifically on the sensor structure, Table 4 shows the typical values, as well as the associated FSR. *L* is associated with the cavity length, λ is the wavelength of the light source, ${\left(\Delta n\right)}_{i}$ (in MZI and MI) is the effective mode index difference between core mode and *i*th secondary mode, while *n* (in FPI) is the refractive index of the cavity. It is noted that, unlike the MZI, in MI and FPI the OPD is associated with traveling twice the cavity (2L) due to its reflection detection mode. However, it is emphasized again in the fact that the propagated modes are considered to travel the same distance. Otherwise, the information in Table 4 would not be valid.

**Figure 11:**
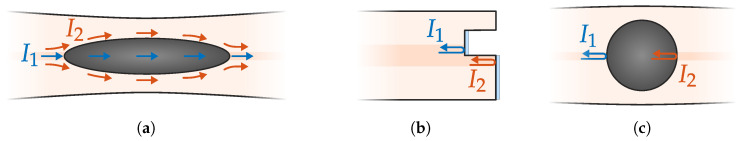
Typical interferometric sensors using in-fiber microcavities. The configurations used are (**a**) MZI, (**b**) Michelson interferometer (MI), (**c**) and Fabry–Perot interferometer (FPI).

Figure 11 shows some typical MZI, MI, and FPI configurations using in-fiber microcavities.

#### 3.3.2. Manufacturing Techniques

Regarding the manufacturing methods of in-fiber microcavities, there are a wide variety of techniques. In the literature, in-fiber air microcavities have been manufactured using silica tubes (hollow-core fiber) [130], photonic crystal fibers (PCFs) [131], chemical etching [132], special fusion splicers [133], and direct processing using femtosecond lasers. Due to the theme of this review, only the last case will be addressed.

The structures that can be manufactured can be classified into two main groups, depending on whether the cavity has access ports with the surrounding medium, or whether it is an air bubble embedded within the fiber. The manufacturing methods may vary depending on what is intended.

**Figure 12:**
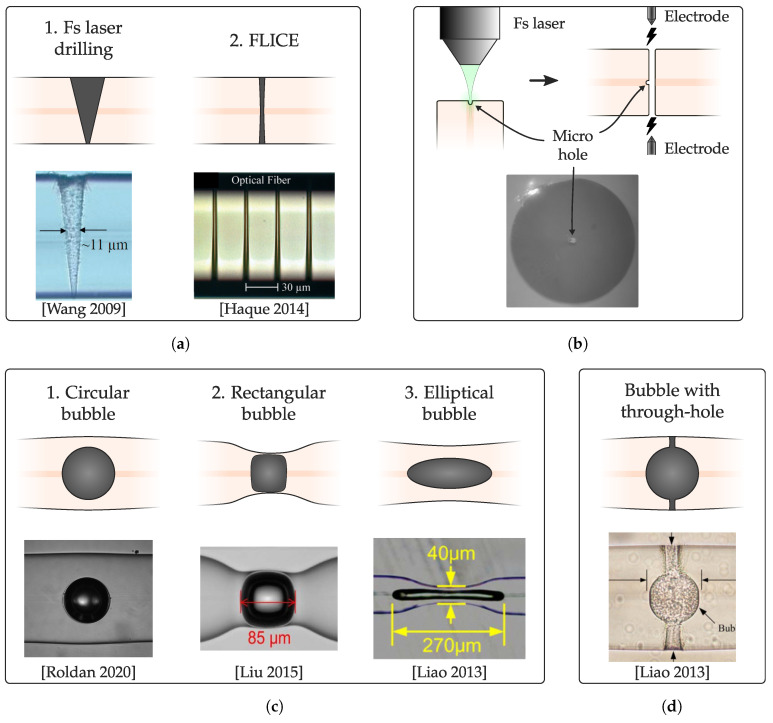
(**a**) Cavities that present access ports with the surroundingmedium. They can bemanufactured by fs laser drilling of optical fiber (adapted with permission from [137], ©The Optical Society) (1), or by the FLICE technique [8] (2). (**b**)Manufacturing process of in-fiber air bubbles by fs laser micromachining of a fiber end-face and subsequent splice [17]. (**c**) Using the technique of Figure 12b, creation of circular bubbles (adapted with permission from [17], ©IEEE) (1), rectangular bubbles by suitable parameters in the splice [133] (2), or bubbleswith great ellipticity through fiber tapering (adaptedwith permission from [135], ©The Optical Society) (3). (**d**) In-fiber bubble with channels to the surrounding medium (adapted with permission from [136], ©The Optical Society). Combination of the methods of Figure 12a,c, in that order.

Within the first group, there are mainly microholes, which can have two access ports (transverse through-hole), or only one. The main characteristic of this type of cavity is its greater sensitivity to changes in the surrounding medium. In this case, there are typically two manufacturing methods. The first of them consists of using the fs laser to drill the fiber (ablation modification) without any subsequent chemical etching procedure, which results in holes with a marked conical morphology, more irregular surface, and superior diameters (Figure 12(a1)). However, its manufacture is relatively simple. The second manufacturing method corresponds to the FLICE technique, already explained in Section 2.3. The obtained microchannels will be exhaustively developed in Section 3.4. First, the fiber is processed with the laser (Type II modification) to be subsequently attacked with a chemical agent such as HF. The holes obtained in this case have a notably higher aspect ratio, a surface with less roughness, but they have the disadvantage of requiring the use of chemical etching (Figure 12(a2)). Using this last method, not only microholes can be manufactured, but more complex structures, such as the one detailed by Yuan et al. in 2014 [128] (Figure 12c).

The second major group corresponds to in-fiber air bubbles, without access ports. The method of manufacturing in-fiber air cavities using fs lasers is based on two steps, depicted in Figure 12b [17]. Two cleaved sections of fiber are required. First of all, in the end-face of one of the sections, a small hole is made using an fs laser. Typically, the microhole is made in the core to have bubbles centered in the fiber, but it can be done anywhere in the end-face if non-centered bubbles are required. The dimensions of the microhole determine the subsequent bubble size, as well as the time and current of the electric arc discharge of the splice. Table 5 shows the values of these parameters according to various works collected in the literature. The generated bubble is, in any case, circular (Figure 12(c1)).

From the circular bubble, it is possible to obtain rectangular cavities, as demonstrated by Liu et al. in 2015 [133] (Figure 12(c2)). In principle, in this work they do not use fs lasers, but only a commercial fusion splicer Fujikura FSM-60S. However, a small circular bubble can be made according to the technique presented in Figure 12b, and continue with the last steps of the technique in [133]. Basically, once the mini-bubble is obtained, a new arc is applied while the left and right fiber holders move backward to each other. In this way, a rectangular bubble can be obtained with a remarkably high sensitivity in parameters such as strain (43 pm/με).

On the other hand, to obtain markedly elliptical cavities from circular bubbles, fiber tapering must be performed using the flame brushing technique [135] (Figure 12(c3)). It should also be noted that it is possible to manufacture in-fiber air cavities and, subsequently, make microchannels in order to have access ports with the surrounding medium [136] (Figure 12d). In this way, since it has an internal cavity with a larger size, the fluidity of the external liquid is greater, increasing the adaptability of the sensor to external changes.

#### 3.3.3. Sensor Structures and Applications

In recent years, the integration of in-line interforemeters in optical fibers has attracted remarkable research interest, due to its applicability in many sensing applications, reduced size (mm-size, or even μm-size), and ease of manufacture, in this case, by micromachining using lasers.

Using microcavities formed from bubbles or transverse microholes, various types of in-fiber interferometer configurations can be made, such as Fabry–Perot, Mach–Zehnder, or Michelson interferometers. Table 6 presents some of the most notable cavity-based optical fiber sensors collected in the literature.

**Figure 13:**
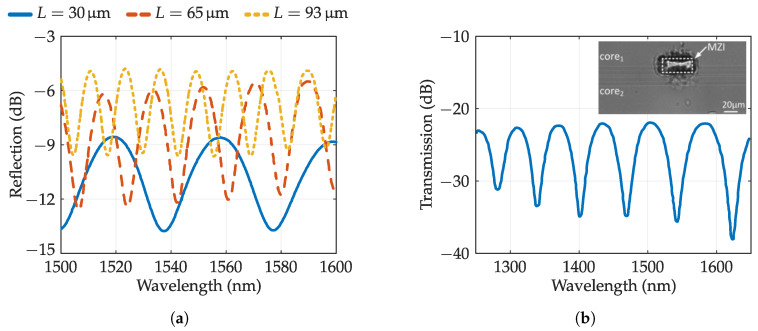
(**a**) Reflection spectra of bubble-based FPI depending on the diameter of the circular bubble, reproduced from [134]. (**b**) Transmission spectrum of an MZI manufactured by means of a through-hole in one core of a twin core fiber, reproduced from [139]. Inset: microscope top-view image of the MZI (adapted with permission from [139], ©The Optical Society).

Fabry–Perot interferometer is perhaps the immediate application of the use of in-fiber microcavities, due to their nature (Figure 11c). As indicated in the table, cavity-based FPI sensors have been made from all the laser manufacturing techniques detailed above, that is, holes manufactured using direct ablation [137,138] or FLICE technique [128], in-fiber bubbles (circular or rectangular) [133], as well as bubbles with through-holes [134]. In Figure 13a, different reflection spectra are depicted depending on the diameter of the circular bubble (cavity length) according to the results obtained by Liao et al. in 2012 [134]. It seems logical that the sensing of the surrounding refractive index (SRI) is one of its main applications, with notably high sensitivities. Moreover, the possibility of depositing metal films on the cavity walls makes it possible to detect, among other things, concentrations of gases such as hydrogen [138].

Another type of interferometer that can be manufactured with in-fiber cavities is the Mach–Zehnder interferometer. There are multiple ways to generate forward OPD with cavities: with markedly elliptical bubbles using tapered fibers (Figure 11a) as demonstrated by Liao et al. in 2013 [135]; with a through-hole that affects only one of the cores of a twin core fiber (Figure 13b), developed by Li et al. in 2015 [139]; using two small circular bubbles at the ends of a tapered fiber, a sensor manufactured by Liao et al. in 2019 [140]; as well as through a cavity (with access ports) that only modifies half of the core, by Liao et al. in 2016 [141]. There are numerous examples in the literature, but Table 6 has sought to expose the most relevant and varied. As a result, there are interference patterns with more transmission losses than counterparts made with waveguides [124]. However, due to the abrupt refractive index change caused by the cavity, the sensors obtained are more compact and with a better fringe visibility of >12 dB. These sensors have been used to sense gas pressure [139], or ethanol concentration [140], among others. In addition, notable improvements in SRI sensitivity have been achieved (−10,223 nm/RIU between 1.3–1.325) [141].

The last type of interferometer addressed is the Michelson, with a reflection detection mode. In 2012, Liao et al. developed a sensor with the configuration depicted in Figure 11b for sensing the surrounding refractive index. It has a cavity length of 38μm (difference in optical path in reflection), and a 90 nm Ag film has been placed on the end-face of the fiber.

Although the main application of in-fiber microcavities is focused on interferometry, optical resonators have also been manufactured in different works. Beyond typical optical resonator configurations, in-fiber bubbles have begun to be used as resonators in recent years by exciting whispering gallery modes (WGM) [144]. In 2018, Liu et al. applied the in-fiber rectangular bubble detailed in [133] to make a micro-optical WGM resonator [127]. The bubble, similar to the one depicted in Figure 12(c2), has a diameter of 78μm along its equator surface and an ultra-thin wall thickness of ∼1 μm. Thus, the manufactured resonator has a quality factor (*Q*) greater than $10^{6}$, and a total strain-based tunable bandwidth of ∼14.12 nm. Microcavity-based WGM resonators have been used in recent years for nanoparticle sensing. In fact, in 2018 Ward et al. detected 100 nm and 500 nm polystyrene particles in aqueous solution [145].

Furthermore, there are also microcavity-based grating optical fiber sensors. In 2013, Guo et al. designed a 3 mm LPG using 100μm separated microchannels manufactured using the FLICE technique [129]. The structure allows to measure SRI, temperature and strain, although not simultaneously.

**Figure 14:**
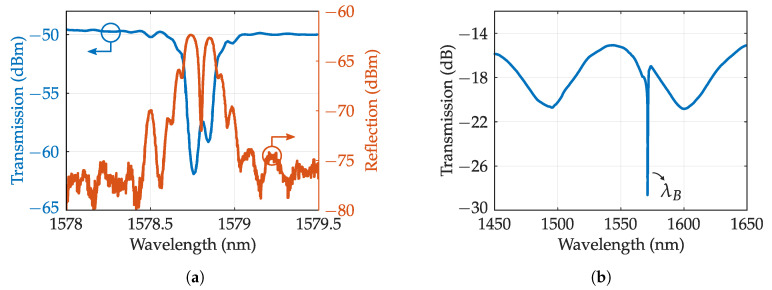
(**a**) Transmission and reflection spectra of bubble-based phase-shifted FBG, reproduced from [136]. (**b**) Transmission spectrum of cavity-based MZI embedded in FBG, reproduced from [143].

To be able to undertake the sensing of multiple parameters simultaneously, at least two sensor structures are required, with different sensitivities to parameters. This makes it possible to limit errors derived from cross-sensitivity. In 2013, Liao et al. manufactured a phase-shifted FBG by including an in-fiber bubble with through-hole like the one shown in Figure 12d. In this way, through variations in temperature, strain or SRI, there is a shift in the cavity resonance peak. As shown by the spectra of the structure depicted in Figure 14a, $\lambda_{B}$ of the FBG as well as the resonance peak of the cavity allow two parameters to be sensed simultaneously.

Ref. [143] also shows a optical fiber sensor that allows multi-parameter sensing. It is an MZI embedded in an FBG. The MZI is based on a 48μm cavity (through-hole) that only affects approximately half of the core, creating the interference depicted in the spectrum of Figure 14b. Variations of its FSR, and $\lambda_{B}$ shifts allow, as in the previous case, to discriminate two parameters simultaneously.

Recently, Roldán et al. have reported a cavity-based optical fiber sensor that allows multi-parameter sensing [17]. It is an ultra-short (<300 μm) LIF sensor integrated into a surgical needle. It is a sensor oriented to biomedical applications: its operating principle follows the *“touch and measure”* approach, allowing the SRI of a fluid to be detected when the sensor contacts a tissue (strain).

### 3.4. Microchannels and Optofluidic Structures

Femtosecond lasers provides the advantage of using the same system for the manufacture of waveguides and microchannels. In this way, it is possible to integrate both structures in the same optical fiber, being able to carry out complex optofluidic devices. To undertake the manufacture of microfluidic channels, the FLICE technique developed in Section 2.3 can be used.

There are two large groups in which the microchannels can be placed. The characteristics of both types are given by the inscription geometry of the fs laser in step 1 of the FLICE technique [20]. If the manufactured microchannel is derived from a fs laser inscription in the same direction as the laser beam propagation direction (longitudinal geometry), as depicted in Figure 2, the channel has a circular cross-section, but its length is limited by the working distance of the objective lens. In case the microchannel is oriented perpendicular to the laser beam propagation direction (transverse geometry), the manufacturing flexibility is notably higher, since channels of arbitrary length and shape can be made. However, the transverse geometry has a great disadvantage: the cross-section of the fs laser irradiated region is really asymmetric, because the Rayleigh distance ($z_{0}$) is greater than the waist radius ($w_{0}$). The relation of proportionality is $z_{0}\propto w_{0}^{2}$ [71]. Accordingly, the cross-section of the focal volume is strongly elliptical in the laser beam propagation direction. Although lower laser wavelengths result in more symmetrical focal volumes, the originated channels will always have an elliptical cross-section, which is undesirable for optofluidic applications.

There are many solutions in the literature that seek to achieve focal volumes with a symmetrical cross-section. First, it seems clear that overlapping different slightly offset laser scans results in a quasi-circular cross section. This is known as the multiscan technique [124], developed in more detail in the waveguides section. Another solution is the astigmatic beam shaping technique, initially proposed by Cerullo et al. (2002) [146]. In this technique, a cylindrical telescope modifies the resulting Rayleigh distance after the microscope objective in order to achieve circular microchannels [55]. Another solution is the slit beam method [147]. Here, the cylindrical telescope is replaced by a simple slit before the objective lens, being more compact and requiring less complexity in optical alignment, but with a higher pulse energy requirement. More solutions can be found in the literature, such as using active optics [148], or spatiotemporal focusing techniques [149].

Next, examples of manufactured microfluidic channels will be presented, with the characteristics that define them; and later, some of the most advanced optofluidic devices reported in the literature will be detailed, with the corresponding application. Both optical and fluidic parts of these sensors have been manufactured using fs laser processing. All presented structures have been manufactured within fused silica.

Table 7 lists some of the most representative microfluidic channels found in the literature that have been manufactured using the FLICE technique. fs laser inscription properties in FLICE step 1 are presented, as well as the characteristics of the chemical etching (step 2). Following the theme of the review, it is intended to make special emphasis on microchannels made within optical fibers. However, some examples carried out in bulk glass are also presented (origin of FLICE technique) in order to explore the differences between them.

The microchannels developed in bulk glass stand out for using transverse geometry, allowing greater flexibility in the creation of optofluidic devices. In this way, they are not limited by the thickness of the bulk, or the lens working distance; and the cross-section asymmetry is corrected with some of the previously mentioned techniques [55,147]. In addition, using HF as an acid, channels with a length of 1.5–2 mm are obtained as the best result [41,55,56,58]. Typically, their aspect ratio (considering that the channel has a conical shape) is between 15 and 20. It is observed that, if a channel is made with 2 access ports, both the length and the aspect ratio of the generated channel can be significantly improved [41]. Likewise, it is also observed that KOH can be used as an alternative to HF for highly selective etching of femtosecond laser irradiated microchannels. Unlike using HF, with KOH no saturation is observed in the microchannel length. The etching selectivity remains practically constant regardless of the etching time. Kiyama et al. (2009), through prolonged etching (60 h with 35.8% KOH solution at 80 °C), obtained channels as long as 1 cm with less than 60μm in diameter (aspect ratio of ∼200) [58].

**Figure 15:**
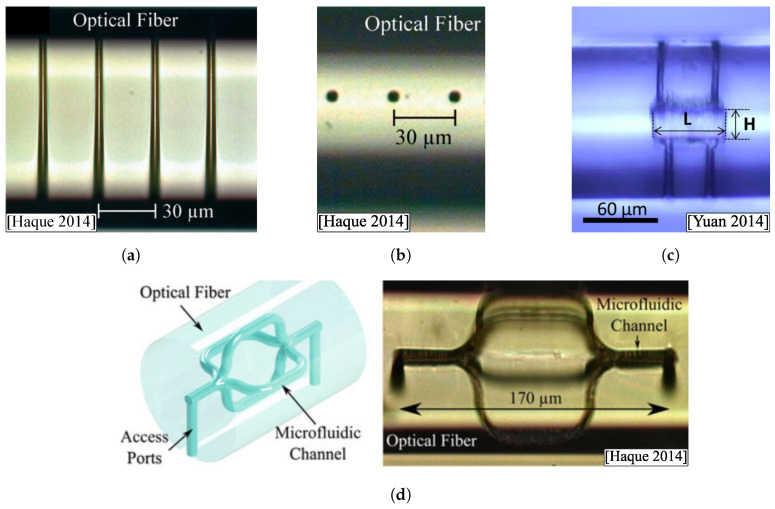
(**a**) Microchannel made in an SMF (lateral view) [8]. (**b**) Top view of the microchannel access ports manufactured in SMF [8]. (**c**) Fabry–Perot cavity manufactured by chemical etching through different access microchannels (adapted with permission from [128], ©The Optical Society). (**d**) Schematic and image of a 3D microfluidic network manufactured within a coreless optical fiber. One microchannel is split into four separate radial arms [8].

However, in optical fibers, HF has traditionally been used as the chemical agent in FLICE step 2. In order to manufacture 3D optofluidic devices accurately, it is necessary to reduce or eliminate spherical aberration and beam-focusing distortion in the fiber. This is performed similarly as in Section 3.2 by high NA oil-immersion objective lenses (tighter focusing) [8,129], or adaptive optics with air objective lenses [128,150,151]. In the latter case, the fiber is surrounded by index-matching oil and a coverslip is placed above it [124,152]. In addition, the longitudinal inscription geometry is preferably used, since it is sought that the core optically interrogates the fluid that surrounds the fiber. Consequently, microchannels transverse to the longitudinal axis of the fiber are intended, with a maximum length equal to the diameter of the fiber (Figure 15a). Taking into account that the channels manufactured are relatively short, and that they generally have 2 microfluidic access ports, it is possible to have very high aspect ratios, of the order of 20–25, that is, microchannel diameters of less than 6μm (Figure 15b) [8,129,150]. Likewise, short channels imply a faster etching time, typically less than one hour. Lower HF concentrations result in channels with a higher aspect ratio, and less roughness [8].

An important aspect to consider when working with standard SMF fibers is the germanium-doped core. It has a much faster intrinsic etching rate that pure fused silica cladding (∼11.5 times faster) [153]. Consequently, concave surfaces often appear in the core [129,151]. This leads to higher insertion losses due to an undesirable negative lensing effect. This is usually solved by performing the microchannels on coreless fibers, and interrogating the microfluidic channel with fs laser inscribed waveguides [8].

Although the microchannels are the homologous structure to the waveguides in the field of fluidics, other fluidic structures can also be made using the FLICE technique. Optofluidic resonators and microfluidic reservoirs are clear examples of advanced and integrated optofluidic structures. By multiplexing and interconnecting different fiber microsystems, it is possible to design and manufacture miniature LIF platforms with highly functional and distributed sensing capabilities. An outstanding example of the above is presented in the work of Haque et al. (2014) [8].

Microfluidic reservoirs and optical resonators are formed by arrays of fs laser modification scans, which are subsequently subjected to chemical etching. In this case, the roughness of the walls is a key point, so both the polarization in the laser inscription and the subsequent etching parameters must be optimized. These cavities can be manufactured with arbitrary shapes and sizes, being able to have great flexibility in the desired spectral response. In many cases, tapered access ports are manufactured to promote the diffusion of external fluid throughout the cavity. An example of inline Fabry–Perot cavity with a 45° taper access ports is provided by Haque et al., with a cavity of 30μm (FSR≃40 nm), maximum reflection values of −10 dB, and reflection fringe contrast of 15 dB. Outstanding values are due to the 12 nm (rms) wall roughness. Yuan et al. developed in 2014 a cavity-based optofluidic resonator with two input (inlets) and output (outlets) microchannels [128]. SRI sensitivity was 1135 nm/RIU (about RI=1.34). The image of this fluidic structure is depicted in Figure 15c (L=55μm, H=20μm).

There are additional structures in the literature. For example, Lee et al. explored novel microchannel designs: microslot channel along the core, microslot channel perpendicular to the core and helical channel around the core [151]. These designs contributed to an improvement in the power sensitivity of the surrounding refractive index (1.55 dB/RIU). Guo et al. made a compact long-period fiber grating using periodic transverse microchannels [129]. A low temperature sensitivity of 9.95 pm/°C from 30 °C to 120 °C was achieved, and RI sensitivity with one order of magnitude larger than that of the conventional LPGs near RI=1.33. Zhou et al. manufactured a 1.2×125×500μm micro-slot across an FBG, which was used as a refractometer to test the RI of oils with a sensitivity comparable to LPG sensors (945 nm/RIU) [152]. Relatively complex 3D structures can also be manufactured, such as the one shown in Figure 15d [8]. The flexibility of inscribing different patterns in the fiber with the fs laser allows creating microchannels in straight, circular, or arbitrarily complex shapes, increasing the applicability of this type of structures.

With the development of LIF sensors, and the multiplexing of optofluidic structures such as those detailed, it is possible to detect a wide set of different parameters. The optical interrogation of fluidic structures allows an extraordinary increase in sensitivity to the surrounding refractive index, but also detects fluorescence, particle separation by capillary electrophoresis, cell sorting, flow cytometry, or cell trapping, among others [8,20,41,47]. Consequently, these types of biophotonic devices are really attractive in fields such as biology or biochemistry, as well as in medical procedures (smart catheters).

### 3.5. Scattering Dots

Optical backscatter detection techniques have traditionally been considered to perform spatially resolved measurements [154]. It is possible to use distributed sensors with a detection range up to 100 km, which makes them suitable for different applications such as structural health monitoring, gas and oil pipeline security of subsea cables monitoring, among others.

Linear Rayleigh sensors are being used in distributed or quasi-distributed sensing domain to measure different parameters, such as distributed vibration sensing (DVS) or distributed acoustic sensing (DAS) [155,156,157,158,159]. One of the reasons is that Rayleigh backscatter signal intensity is higher than that of their non-linear Brillouin and Raman counterparts.

It is worthy to recall that Rayleigh scattering occurs as a consequence of the randomly distributed refractive index fluctuations due to the intrinsic nature of the glasses that constitute the fibers [154]. The randomness of the scattered distribution (and fading) leads to a quasi-random nonlinear fiber transfer function, which implies that the parameters can only be detected but not quantified [159]. These reflectors, characterized by a very small reflection, nevertheless have a backscatter signal contribution that is dominant compared to that of the fiber itself. For example, in technologies that require phase information, it is possible to take advantage of the dominant pattern caused by the quasi-distributed Fabry–Perot interference between the backscatter waves from each two weak reflectors. While OTDR is currently the most widely used type of reflectometer when working with such weak reflectors [156,157,158], there are also configurations that use OFDR for sensing [155].

**Figure 16:**
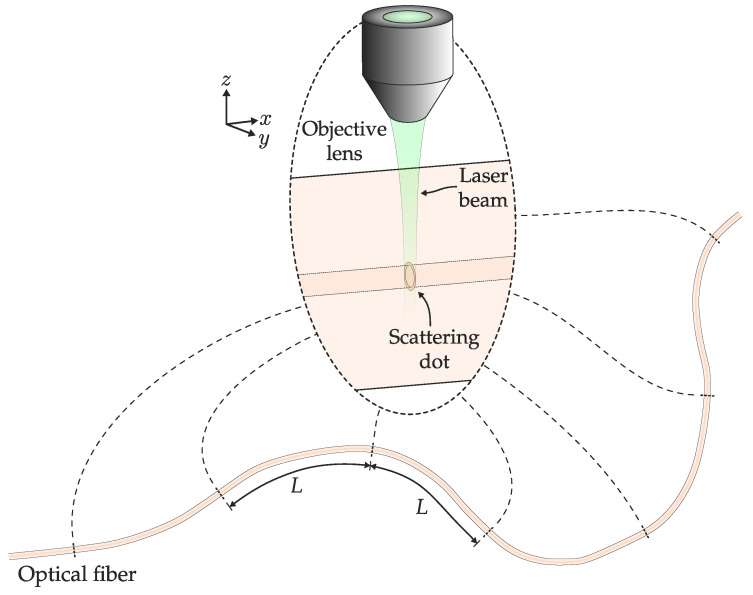
Illustrative diagram of several weak reflectors (scattering dots) located within an optical fiber, and equally spaced distance *L* between each pair. The refractive index changes have been generated from the tight focusing of femtosecond laser pulses.

Regarding the characteristics of the reflectors, many types have been used, such as regular FBGs, weak FBGs, chirped FBGs (CFBGs), fusion splices, in-line splices, or fiber connectors. However, in recent times, there has been a notable growth in the manufacture of scatter centers in the fiber core by using focused fs laser pulses, as depicted in Figure 16.

In 2013, Liehr et al. used for the first time a femtosecond laser to increase the Rayleigh backscatter signal compared to that of the unmodified regions of the fiber [156]. Specifically, they induced voids in the core of a POF. It can be deduced that the reflected signal improved remarkably. However, the losses were so relevant that it was impossible to have a sensor fiber with a length greater than a few meters.

In later works, such as those developed by Donko et al. in the last two years, it has been shown that it is possible to generate fs laser-based weak reflectors (reflecting ∼$2.5\times 10^{-5}\%$) that improve by two orders of magnitude Rayleigh backscatter signal in comparison to unmodified parts of the SMF fiber while maintaining minimal losses [157,158]. By means of smooth Type I RICs, equally spaced reflectors were arranged every 1 m, giving rise to losses of 0.3 dB/km, which makes it possible to have sensor fibers of tens and even hundreds of km.

In 2019, Hicke et al. conducted a study on the use of fs laser point-by-point scattering dots [159]. By varying the inscription process parameters, they created weak reflectors with flexible control of their reflectivity and attenuation, maintaining a linear fiber transfer function.

By using femtosecond laser technology, dots with a pseudo-random pitch have been inscribed in a single-mode fiber. With this approach, a quasi-randomly distributed reflector (working as a sensing mirror) in a short-cavity fiber optic laser sensor has been reported [160,161]. A better sensitivity for the measurement of strain than regular FBG transducers has been offered by this fiber laser sensor.

## 4. Conclusions

The most remarkable transducer structures conceived in optical fibers by laser processing have been highlighted. Laser processing allows, in addition to traditional ablation, permanent RIC, film deposition, and selective material removal through FELICE. With these tools, it is possible to manufacture gratings, waveguides, cavities, microchannels, and reflectors. These elements, when strategically combined, can create new fiber-integrated devices that combine multiple sensor elements, that is, Lab-in-Fiber sensors.

FBGs are highly versatile and technologically mature key structures that can still be further improved by the direct-write method assisted by proper beam shaping techniques. The proper cross-sectional design of FBGs can be an effective way to design polarization-dependent cladding mode assisted sensors. In addition, there is a growing effort to achieve stable FBGs at extreme temperatures, in these cases, rFBGs and fs-IR FBGs are considered solid candidates.

Waveguide inscription technology in optical fibers has shown promising results and first fiber interferometers exhibit interesting properties. Furthermore, these structures are of special interest to develop compact multi-parameter sensors through several interconnected cladding structures. However, there is still a need to develop optimized schemes to achieve optimal results. In particular, in addition to the improved waveguide cross-sections, efficient coupling of the fiber core to the CWG remains one of the main challenges for these structures.

Microchannels and large cavities are of special interest for several optofluidic applications, being remarkably studied in fields such as biology, chemistry, biomedicine, as well as clinical procedures. Microchannels inside the fiber can be manufactured directly by laser ablation, or by means of the FLICE technique, which, thanks to the chemical agent, allows having microchannels with significantly higher aspect ratios. Microcavities, by the other way, can also be manufactured by other techniques, such as laser ablation, use of special fibers, or by combining laser and splice processing, among others. In these cases, in-fiber bubbles are usually generated, which can present different geometries (circular, rectangular, or elliptical) after the corresponding post-processing. They are typically used as interferometers, in some of their different configurations, and can be used in combination with other sensor structures for multi-parameter sensing.

Finally, the increase of Rayleigh backscattering in optical fibers can be achieved by weak reflectors or scattering dot inscription typically manufactured using femtosecond lasers. They are useful for both fiber sensing and fiber laser structures. The latest works in the literature offer an improvement of two orders of magnitude of the Rayleigh backscatter signal in comparison to unmodified parts of the fiber while maintaining minimal losses.

The reader is encouraged to delve into the topics shown in this work. For that purpose, Table 8 depicts a list of useful reviews that cover several points in greater detail.

## Figures and Tables

**Table 1:** List of different gratings and some of their properties.

FBG Type	Subcategory	Characteristics
Type I	Type I UV	∘ Written in photosensitive fibers with phase mask
		∘ Complete erasure at 600–800 °C [64]
	Type I fs-IR	▹ Smooth RIC caused by defects centers, small $T_{f}$ modification and low stress field
		▹ It can resist up to 1000 °C with some erasure [64,65], similar to Type II
		▹ Homogeneous RIC, commonly written with phase mask
	⋯	⋄ A broader list can be found in [66]
Type II	Type II UV	∘ Excimer lasers are typically employed
		∘ High thermal resistance and scattering losses
	Type II fs-IR	▹ In PbP: permanent RIC and/or microvoids structure
		▹ In phase-mask: nanogratings (Type II)
		▹ Inhomogeneous RIC and high scattering losses
		▹ Stable above 1000 °C with little degradation [67]
Type R		∘ Erasure and resurrection of UV or fs seed FBG
		∘ Stable up to 1295 °C with losses comparable to Type I [63,66]

**Table 2:** Inscription parameters of cladding waveguides (CWG) in literature.

Ref.	Inscription Method	NA	λ (nm)	PRR (kHz)	$\frac{\mathrm{pulses}}{\mu m}$	τ (fs)	$E_{p}$ (μJ)
[120]	Oil immersion	×100,1.25	800	5	500	35	0.5
[119]	Oil immersion	×60,1.4	532	200	1000	250	0.12
[121]	Oil immersion	×100,1.25	522	1000	10,000	230	0.071
[112]	Multiscan	×100,0.42	517	50	1000	220	0.11
[122]	Multiscan	×100,0.5	1030	60	800	370	0.19
[114]	Multiscan	×20,0.4	800	5	−	80	0.14
[118]	Filamented waveguide	×100,0.5	1030	120	1200	370	1.09

**Table 3:** Measured parameters with CWGMZIs in literature.

Ref.	Temperature	Curvature	Strain	RI
[125]	Non linear (50–650 °C)	−	−	−
[120]	0.22 nm/°C (20–90 °C)	−	4.89pm/μϵ	−
[119]	0.6nmfluctuationin 25–100 °C	−	−	$60\;\mathrm{nm}/\mathrm{RIU}\;{}^{a}$
[112]	0.49nm/°C (50–60 °C)	(with FBG)	(with FBG)	(with FBG)
[118]	0.023nm/°C (25–180 °C)	$9.49\;\mathrm{nm}/m^{-1}$	−	−

${}^{a}$ RI Range: 1.33–1.34.

**Table 4:** Typical phase delay ($\delta_{i}$) and Free Spectral Range (FSR) depending on the interferometer configuration used.

Parameter	MZI	MI	FPI
$\delta_{i}$	$\frac{2\pi {\left(\Delta n\right)}_{i}\cdot L}{\lambda }$	$\frac{2\pi {\left(\Delta n\right)}_{i}\cdot 2L}{\lambda }$	$\frac{2\pi n\cdot 2L}{\lambda }$
FSR	$\frac{\lambda^{2}}{\Delta n\cdot L}$	$\frac{\lambda^{2}}{\Delta n\cdot 2L}$	$\frac{\lambda^{2}}{n\cdot 2L}$

**Table 5:** Different manufacturing parameters of in-fiber circular air cavities according to the method depicted in Figure 12b.

Ref.	fs Laser Pulse Energy/Power	Microhole Diameter	Fusing Current	Fusing Duration	Bubble Diameter
[17]	2.7μJ	∼3 μm	25% max.	1.5 s	72μm
[134]	2μJ	∼1 μm	16.3 mA	2 s	60μm
[134]	2μJ	∼1 μm	15.3 mA	1.5 s	30μm
[134]	2μJ	∼1 μm	16.8 mA	2 s	65μm
[134]	2μJ	∼10 μm	17.3 mA	2 s	93μm
[135]	5 mW	∼2 μm	16.3 mA	2 s	65μm
[136]	1 mW (PRR=1 kHz, 1 s)	−	16.3 mA	2 s	50μm

**Table 6:** Most relevant microcavities-based optical fiber sensors existing in the literature and manufactured using lasers.

Ref.	Optical Structure	Microcavity Type	Parameter	Sensitivity	Characteristics
[128]	FPI	Microcavity	SRI	1135.7 nm/RIU	∘ Cavity 55×20μm, ∅hole 5μm
		with holes		(1.333–1.347)	∘ FLICE: 20% HF, 10 min
[133]	FPI	Rectangular	Strain	43 pm/με	∘ Bubble diameter: 61μm
		bubble			∘ Allows WGM resonator [127]
[134]	FPI	Bubble with	SRI	994 nm/RIU	∘ ∅(bubble, holes): (60, 30μm)
		through-hole		(1.31–1.39)	∘ Different bubble sizes studied
[137]	FPI	Microhole	SRI	110 dB/RIU	∘ SRI resolution: $6.7\times 10^{-5}$
		(direct ablation)		(1.37–1.42)	∘ Conical shape (∅8μm at core)
[138]	FPI	Pd coated	Hydrogen	2.45 nm/vol	∘ Cavity length: 20μm
		through-hole	concentr.	(2–8%)	∘ 20 nm Pd film
[135]	MZI	Elliptical	Strain	6.8 pm/με	∘ Major axis bubble: 860μm
		bubble			∘ Waist ∅ tapered fiber: 23μm
[139]	MZI	Through-hole in	Gas	−9.6 nm/MPa	∘ Microchannel width: 45μm
		twin core fiber	pressure	(0–2 MPa)	∘ Direct ablation
[140]	MZI	2 circular bubble	Ethanol	28 nm/vol	∘ ∅bubbles: 41 and 45μm
		in tapered fiber	concentr.	(30–70%)	∘ Separation bubbles: 350μm
[141]	MZI	Half core cavity	SRI	−10,223nm/RIU	∘ Cavity (sensing arm): 93μm
		with access ports		(1.3−1.325)	∘ 4 microchannels (access ports)
[142]	MI	Ag coated	SRI	975 nm/RIU	∘ Cavity length: 38μm
		end-face cavity		(1.46–1.492)	∘ 90 nm Ag film
[129]	LPG	Microholes	SRI	−692 nm/RIU	∘L=3mm,Λ=100μm
		(FLICE)	Temp.	9.95 pm/°C	∘ FLICE: 4% HF, 20 min
			Strain	−2.4 nm/mε	
[136]	Phase-shifted	Bubble with	SRI	9.9 nm/RIU	∘ Multi-parameter sensing 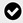
	FBG (bubble	through-hole	Temp.	10.2 pm/°C	∘ Tunable phase-shifting
	in middle)		Strain	0.481 pm/με	∘ Bubble diameter: 50μm
[143]	MZI	Half core	SRI	−9148 nm/RIU	∘ Multi-parameter sensing 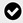
	embedded	through-hole		FBG: 12 pm/°C	∘ Cavity length: 48μm
	in FBG		Temp.	MZI: 15 pm/°C	∘ Direct ablation
[17]	FPI with	Circular	SRI	11.5 nm/RIU	∘ Multi-parameter sensing 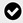
	Pl-b-Pl FBG	bubble	Strain	6.69 pm/με	∘ Cavity length: 73μm
					∘ Integrated in surgical needle

**Table 7:** Fs laser inscription parameters (step 1) and chemical etching properties (step 2) of microfluidic channels in literature. Their length and aspect ratio are indicated. The shaded rows correspond to microchannels in optical fibers.

Ref.	NA Lens	Geometry	PRR (kHz)	Speed (μm/s)	$E_{p}$ (μJ)	Acid	Conc.	Etching Time (h)	Length (mm)	Aspect Ratio	Access Ports
[41]	0.6	Transversal	1	20	3	HF	15%	3.5	1.8	20	1
[41]	0.6	Transversal	1	20	3	HF	15%	3.5	3	33.3	2
[55]	0.6	Transversal	1	20	3	HF	15%	3.5	1.5	18.7	1
[56]	0.65	Transversal	100	30	1	HF	2.5%	8	2.1	−	1
[56]	0.45	Longitudinal	100	110	0.2	HF	2.5%	5	1.2	−	1
[58]	0.65	Transversal	1	100	0.36	HF	2%	48	1.9	17.7	1
[58]	0.65	Transversal	1	100	0.36	KOH	35.8%	60 (80 °C)	∼1000	∼200	2
[8]	1.25	Longitudinal	1000	300	0.03	HF	5%	1	0.125	25	2
[128]	0.4	Longitudinal	250	100	0.4	HF	20%	10 min	0.125	19	2
[150]	0.55	Longitudinal	1	−	0.2	HF	15%	15 min	0.125	25	2
[151]	0.5	Longitudinal	100	−	0.1	HF	5%	25 min	0.125	22.3	2
[129]	1.42	Longitudinal	1	−	−	HF	4%	19 min	0.125	25	2

**Table 8:** List of selected further readings to delve into the topics discussed in this work.

Ref.	Year	Structure	Description
[71]	2012	FBG	Properties of FBG inscribed by femtosecond lasers
[66]	2008	FBG	Extensive list of FBG types
[76]	2017	FBG	Polarization assisted FBG sensors
[47]	2015	CWG	In-depth guide of oil inmersion CWGs
[7]	2012	Cavities	Interferometric cavity-based optical fiber sensors
[41]	2011	Microchannels	fs laser manufacturing of optofluidic devices
[50]	2019	Microchannels	fs nanoprocessing of 3D structures
[61]	2019	FBG	Optical Fiber Grating-Based Plasmonic Sensors
[162]	2019	Several	Multi-parameter fiber-optic sensors
[163]	2019	Several	Beam shaping through adaptive optics
[31]	2011	–	Study of different fs IR modification threshold in pure silica

## Abbreviations

The following abbreviations are used in this manuscript:

CFBG	Chirped Fiber Bragg Grating
CWG	Cladding Waveguide
CWGMZI	Cladding Waveguide Mach–Zehnder Interferomer
DAS	Distributed Acoustic Sensing
DVS	Distributed Vibration Sensing
FBG	Fiber Bragg Grating
FLICE	Femtosecond Laser Irradiation followed by Chemical Etching
FPI	Fabry–Perot Interferometer
FSR	Free Spectral Range
GODC	Germanium Oxygen-vacancy Defect Center
KKr	Kramers–Konig relations
LbL	Line-by-Line
LIF	Lab-In-Fiber
LIRIC	Laser Induced Refractive Index Change
LOC	Lab-On-Chip
LPG	Long-Period Grating
MI	Michelson Interferometer
MZI	Mach–Zehnder Interferometer
NA	Numerical Aperture
NBOHC	Non-Bridging Oxygen Hole Center
OFS	Optical Fiber Sensor
OPD	Optical Path Difference
PbP	Point-by-Point
PCF	Photonic Crystal Fiber
PIC	Photonic Integrated Circuit
Pl-b-Pl	Plane-by-Plane
PDL	Polarization Dependent Loss
POF	Polymer Optical Fiber
PRR	Pulse Repetition Rate
QPM	Quantitative Phase Microscopy
RI	Refractive Index
RIC	Refractive Index Change
RIU	Refractive Index Unit
RNF	Refracted Near Field
SEM	Scanning Electron Microscope
SMF	Single Mode Fiber
SRI	Surrounding Refractive Index
TFBG	Tilted Fiber Bragg Grating
TOF	Tapered Optical Fiber
WBG	Waveguide Bragg Grating
WGM	Whispering Gallery Mode
